# Evaluation of the SUCCESS Health Literacy App for Australian Adults With Chronic Kidney Disease: Protocol for a Pragmatic Randomized Controlled Trial

**DOI:** 10.2196/39909

**Published:** 2022-08-31

**Authors:** Jennifer Isautier, Angela C Webster, Kelly Lambert, Heather L Shepherd, Kirsten McCaffery, Kamal Sud, Jinman Kim, Na Liu, Nicole De La Mata, Shahreen Raihana, Patrick J Kelly, Danielle M Muscat, SUCCESS Study Group

**Affiliations:** 1 Sydney Health Literacy Lab, Sydney School of Public Health Faculty of Medicine and Health The University of Sydney Sydney Australia; 2 Sydney School of Public Health Faculty of Medicine and Health The University of Sydney Sydney Australia; 3 National Health and Medical Research Council Clinical Trials Centre The University of Sydney Sydney Australia; 4 Westmead Applied Research Centre Westmead Hospital Westmead Australia; 5 School of Medicine Faculty of Science, Medicine and Health University of Wollongong Wollongong Australia; 6 Illawarra Health and Medical Research Institute Wollongong Australia; 7 Centre for Medical Psychology and Evidence-based Decision-making School of Psychology, Faculty of Science The University of Sydney Sydney Australia; 8 Susan Wakil School of Nursing Faculty of Medicine and Health The University of Sydney Sydney Australia; 9 Nepean Clinical School Faculty of Medicine and Health The University of Sydney Sydney Australia; 10 Department of Renal Medicine Nepean Hospital Sydney Australia; 11 School of Computer Science The University of Sydney Sydney Australia; 12 The University of Sydney Business School The University of Sydney Darlington Australia; 13 See Acknowledgments

**Keywords:** chronic kidney disease, health literacy, shared decision-making, eHealth, smartphone app

## Abstract

**Background:**

We developed a smartphone app—the SUCCESS (Supporting Culturally and Linguistically Diverse CKD Patients to Engage in Shared Decision-Making Successfully) app—to support Australian adults with kidney failure undertaking dialysis to actively participate in self-management and decision-making. The content of the SUCCESS app was informed by a theoretical model of health literacy that recognizes the importance of reducing the complexity of health information as well as providing skills necessary to access, understand, and act on this information.

**Objective:**

The purpose of this study is to investigate the efficacy of the SUCCESS app intervention.

**Methods:**

We designed a multicenter pragmatic randomized controlled trial to compare the SUCCESS app plus usual care (intervention) to usual care alone (control). A total of 384 participants receiving in-center or home-based hemodialysis or peritoneal dialysis will be recruited from six local health districts in the Greater Sydney region, New South Wales, Australia. To avoid intervention contamination, a pragmatic randomization approach will be used for participants undergoing in-center dialysis, in which randomization will be based on the days they receive hemodialysis and by center (ie, Monday, Wednesday, and Friday or Tuesday, Thursday, and Saturday). Participants undergoing home-based dialysis will be individually randomized centrally using simple randomization and two stratification factors: language spoken at home and research site. Consenting participants will be invited to use the SUCCESS app for 12 months. The primary endpoints, which will be assessed after 3, 6, and 12 months of app usage, are health literacy skills, evaluated using the Health Literacy Questionnaire; decision self-efficacy, evaluated using the Decision Self-Efficacy Scale; and rates of unscheduled health encounters. Secondary outcomes include patient-reported outcomes (ie, quality of life, evaluated with the 5-level EQ-5D; knowledge; confidence; health behavior; and self-management) and clinical outcomes (ie, symptom burden, evaluated with the Palliative care Outcome Scale–Renal; nutritional status, evaluated with the Patient-Generated Subjective Global Assessment; and intradialytic weight gain). App engagement will be determined via app analytics. All analyses will be undertaken using an intention-to-treat approach comparing the intervention and usual care arms.

**Results:**

The study has been approved by Nepean Blue Mountains Human Research Ethics Committee (2020/ETH00910) and recruitment has begun at nine sites. We expect to finalize data collection by 2023 and publish the manuscript by 2024.

**Conclusions:**

Enhancing health literacy skills for patients undergoing hemodialysis is an important endeavor, given the association between poor health literacy and poor health outcomes, especially among culturally diverse groups. The findings from this trial will be published in peer-reviewed journals and disseminated at conferences, and updates will be shared with partners, including participating local health districts, Kidney Health Australia, and consumers. The SUCCESS app will continue to be available to all participants following trial completion.

**Trial Registration:**

Australia New Zealand Clinical Trials Registry (ANZCTR) ACTRN12621000235808; https://www.anzctr.org.au/Trial/Registration/TrialReview.aspx?id=380754&isReview=true

**International Registered Report Identifier (IRRID):**

DERR1-10.2196/39909

## Introduction

Chronic kidney disease (CKD) affects up to 10% of the Australian population [1], with greater prevalence, higher mortality, and more rapid disease progression in culturally and linguistically diverse communities [2]. The long-term management of CKD is complex, requiring patient involvement both in decision-making and self-management. Effective self-management and health decision-making requires the ability to understand and use health information, a skill that is known as “health literacy” [3]. However, limited health literacy is common in CKD populations [4] and is independently associated with missed dialysis treatments, increased emergency department visits, increased mortality, and poorer quality of life [5,6]. This is further compounded by the fact that cognitive impairment is more prevalent in those undertaking dialysis compared to the general population, with deficits in attention, memory, and executive function [7]. These cognitive deficits may impact cognitive processing, speed, memory, and the ability to plan ahead and strategize—all necessary skills for participating in self-management and decision-making.

Interventions that focus on developing health literacy skills may have positive effects on shared decision-making and self-management for people living with chronic diseases [8]. In people with CKD, there is mounting evidence that health literacy interventions may increase kidney disease–related knowledge, improve self-care behaviors and self-efficacy, and decrease hospitalization rates and length of stay [9]. However, a recent systematic review highlighted that the quality of the evidence is low and that no studies have specifically targeted populations with low health literacy [9]. The authors concluded that future studies should use validated measures of health literacy and determine whether health literacy interventions redress inequity and, specifically, whether these interventions are more beneficial in those with low health literacy [9].

Health literacy interventions may take several forms, such as educational methods varying from formal classes, home visits, and study circles, through to multimedia and eHealth or online interventions [9,10]. eHealth interventions are potentially useful methods to deliver health literacy interventions. It is estimated that over 91% of Australians own a smartphone, and smartphones are used to access information more than any other devices [11]. Furthermore, smartphone interventions can reduce the cognitive burden placed on users by providing small unit-based learning called “microlearning,” allowing users to return to the content at their own pace and in their own time. eHealth interventions have been shown in different settings to provide consumers access to relevant health information, enhance quality of care, and encourage behavior change [12]. Several CKD-related apps already exist; however, current evidence suggests that many CKD-related apps lack accurate, evidence-based information [13], and none are designed based on theoretical models to improve health literacy.

We recently developed a smartphone app—the SUCCESS (Supporting Culturally and Linguistically Diverse CKD Patients to Engage in Shared Decision-Making Successfully) app—to support Australian adults with kidney failure to actively participate in self-management and decision-making [14]. The SUCCESS app was developed with a multidisciplinary team that included researchers in various fields (ie, health literacy, shared decision-making, public health, epidemiology, and computer science), renal clinicians (ie, nephrologists, dietitians, physiotherapists, and social workers), and consumers living with kidney failure. Content was informed by a theoretical model of health literacy [15] that recognizes the importance of reducing the complexity of health information as well as supporting consumers to develop their skills and to access, understand, and act on this information. In this way, it adopts strategies to reduce the complexity of the content (ie, diet, fluids, medicine, physical activity, emotional well-being, and supportive care) and includes features to improve the health literacy skills of patients. The latter includes question prompt lists and evidence-based volitional help sheets to support question-asking and behavioral change as well as animated skills training related to communication, shared decision-making, and critical appraisal of health information. Full details of the development and content of the app have been published elsewhere [14].

The purpose of this randomized controlled trial (RCT) is to investigate the efficacy of the SUCCESS app intervention for adults receiving in-center hemodialysis, home-based hemodialysis, or peritoneal dialysis, including those from culturally and linguistically diverse backgrounds and with lower health literacy. Our objectives are to assess the impact of the SUCCESS app on the primary outcomes, including health literacy skills, decision self-efficacy, and rates of unscheduled health encounters, and on the secondary outcomes, including knowledge, confidence, quality of life, symptom burden, and nutritional status.

## Methods

### Overview

The study methods have been informed by a feasibility study of the intervention [16]. This protocol is reported in accordance with the SPIRIT (Standard Protocol Items: Recommendations for Interventional Trials) guidelines and the CONSORT (Consolidated Standards of Reporting Trials) guidelines [17]. The trial was registered on the Australia New Zealand Clinical Trials Registry (ACTRN12621000235808).

### Design

The SUCCESS trial is a 12-month multicenter pragmatic RCT with a 1:1 allocation to two groups: SUCCESS app plus usual care versus usual care alone.

### Intervention

#### Intervention Development

The SUCCESS app was designed using strategies to reduce the complexity of the content as well as to include features to improve the health literacy skills of patients. The app embeds informational content relevant to living with kidney failure covering diet, fluids, medicine, physical activity, emotional well-being, and supportive care [14]. A four-step process was used to simplify written content, including calculating readability statistics, applying the Patient Education Materials Assessment Tool [18], supplementing written information with video and audio content, and incorporating microlearning and interactive quizzes.

The app also includes features to improve the communicative and critical health literacy skills of patients across four domains: skills to (1) access, (2) understand, (3) appraise, and (4) use health information to make health decisions and be involved in self-management. This is achieved through the inclusion of question prompt lists, volitional help sheets, and animated skills training in communication, shared decision-making, and critical appraisal of online information (Textbox 1). The SUCCESS app is designed for use on both iOS and Android platforms.

**Features of the SUCCESS app.**

**Question prompt lists**

- Question prompt lists are prepared lists of questions that are designed to enable patients to identify questions they wish to ask the health care professional.
- Question prompt lists are embedded within the content areas of the SUCCESS (Supporting Culturally and Linguistically Diverse CKD Patients to Engage in Shared Decision-Making Successfully) app (eg, there is a question prompt list related to taking medications).
- Users are able to choose from a predefined list or write their own questions, which are saved in the app dashboard for use in upcoming health care consultations.

**Volitional help sheets**

- A volitional help sheet is a tool designed to enable the construction of effective implementation intentions in which participants are asked to link in memory temptations (ie, difficult situations that result in urges to engage in a specific behavior) with processes of change (ie, means by which behavior is changed or sustained).
- Volitional help sheets are embedded within each content area of the SUCCESS app. For example, participants can create a volitional help sheet to make a plan to drink less fluids. Plans are saved in the app dashboard for future use.

**Videos**

- Animated videos are available to build skills in the following areas:
  - Health care communication (eg, “Talking to your health care team”)
  - Shared decision-making (eg, “Making decisions”)
  - Critical appraisal of online information (eg, “Can I trust this health information?”).

**Quizzes**

- Content-specific quizzes are embedded into the app to encourage feedback and learning through the use of targeted questions separated by levels of difficulty.
- Users receive feedback and further information about the content of the quiz based on their understanding.

#### Public and Patient Involvement

A key strength of the development of the SUCCESS app was the combined effort of clinicians, health literacy experts, and consumers in the app development process. One consumer and a representative of Kidney Health Australia, the largest consumer advocacy organization for people with kidney disease, were part of the development team and provided valuable feedback regarding app content and usability. Further to this, the app was iteratively improved based on consumer feedback during the development the feasibility testing phases leading up to this trial. As part of this trial, we will continue to seek further feedback from participants on the SUCCESS app and make improvements, if required, after completion of the trial.

### Study Setting

This study will be conducted across six local health districts charged with delivery of health care within the public sector in the Greater Sydney region—Sydney, Northern Sydney, Western Sydney, South Western Sydney, Nepean Blue Mountains, and Illawarra Shoalhaven—including six university teaching hospitals and six community dialysis centers. The Greater Sydney region includes 4,823,991 people (64.5% of the New South Wales population; 20% of the Australian population), of which 38% speak a language other than English at home [19]. The research sites were purposively selected to include broad geographical coverage across Sydney as well as socioeconomic and cultural diversity.

### Study Population

The target population for this study are adults aged 18 years or over who are receiving dialysis, including those receiving in-center hemodialysis, either in the hospital or community hemodialysis centers; home hemodialysis; and peritoneal dialysis. Only participants who have the ability to speak English sufficiently well to provide informed consent will be recruited. We intend to translate and culturally adapt the app into other languages to further expand the diversity of future app users. Participants lacking the cognitive capacity to consent as determined by the nursing staff will be excluded.

### Recruitment

Eligible participants will be identified by their health care providers and approached by the research team either in person during dialysis sessions or clinic visits or via telephone, email, or a letter of invitation; at this time, they will be provided with information about the study. Participants will be entered into the study after written consent is obtained on paper or via REDCap (Research Electronic Data Capture) and after eligibility criteria are met.

### Randomization

#### Overview

Participants will be randomly allocated to either the SUCCESS app plus usual care (intervention) or usual care alone (control). Participants who are randomized to the intervention will be invited to use the SUCCESS app for a maximum of 12 months. Participants will be instructed to engage with the app *ad libitum* and will also receive monthly reminders to use the app via push notifications.

We will not randomize all participants individually due to the risk of intervention contamination, where the intervention is experienced by control group members as well as those allocated to the intervention group. This is possible because participants in the control and intervention arms may undergo dialysis in the same room three times each week for several hours. Instead, a two-tier pragmatic approach will be used to randomize participants depending on whether they are undergoing dialysis at home or receiving dialysis at the in-center (ie, satellite) dialysis units. Our planned approach will minimize contamination and allow for within-center comparisons.

#### Dialysis at In-Center Units

It is standard practice for patients on hemodialysis at the in-center units to receive dialysis treatment three times a week. Patients receiving in-center dialysis always attend on the same days: either Mondays, Wednesdays, and Fridays or Tuesdays, Thursdays, and Saturdays. Therefore, a pragmatic approach will be used at each center by randomizing patients according to their pattern of attendance. Depending on the center, all participants attending Monday, Wednesday, and Friday will be assigned to the same study intervention group, and those attending Tuesday, Thursday, and Saturday will be assigned to the alternate study intervention group.

#### Dialysis at Home

Participants will be randomized individually using minimization and two stratification factors: the language spoken at home and the research site. An interactive voice response system will be used for randomization; this allows for research staff to call a third party—the National Health Medical Research Council Clinical Trials Centre—which allocates the randomization order.

### Timeline

**Figure 1:**
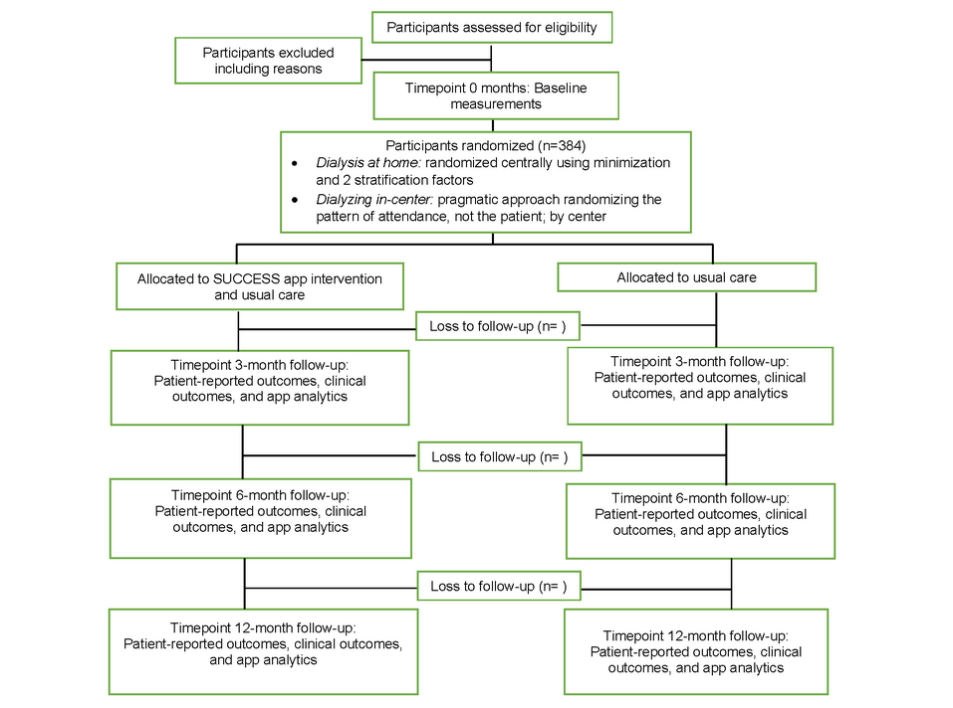
Study flow and timeline. SUCCESS: Supporting Culturally and Linguistically Diverse CKD Patients to Engage in Shared Decision-Making Successfully.

The study timeline will include four data collection timepoints: baseline and 3-month, 6-month, and 12-month follow-ups (Figure 1).

### Study Outcomes

Once informed consent is obtained, baseline measures will be collected, including demographics, dialysis history, cognition level, and health and digital literacy status (Table 1 [20-30]). Our primary outcomes are health literacy, shared decision-making, and rates of unscheduled health encounters; these will be assessed at 3, 6, and 12 months of app usage (Table 1). Secondary outcomes include patient-reported outcomes and clinical outcomes; these will also be assessed at 3, 6, and 12 months of app usage (Table 1).

**Table 1:** Measurements collected throughout the trial.

Measure and description			Timepoint (month)							
			0		3		6		12	
**Baseline measurements**										
	Demographics: age, gender, country of birth, language spoken at home, Aboriginal and Torres Strait Islander status, and highest education attained.	✓^a^								
	Dialysis history: mode of dialysis (hemodialysis or peritoneal dialysis), location of treatment (home or in-center), dialysis schedule (frequency of dialysis per week), and dialysis vintage (years on dialysis).	✓								
	Cognition impairment: the MoCA^b^ is a screening tool developed to assist clinicians in the detection of cognitive impairment [20]. It measures a person’s orientation in time and space, short-term memory, abstract reasoning, attention, and many other aspects of cognitive ability. The MoCA tool was chosen as it is a more sensitive tool for detecting mild cognitive impairment compared to the MMSE^c^ [21]. The MoCA returns a cognitive ability score ranging from 0 to 30, where a score of ≤26 suggests cognitive impairment [20].	✓								
	Health literacy, self-report: the brief health literacy screener is a single-item screener to identify people with inadequate levels of functional health literacy in clinical settings [22,23]. The item asks, “How confident are you filling out medical forms by yourself?”; the item is rated on a 5-point response scale ranging from “not at all” to “extremely.” The threshold for inadequate health literacy is “somewhat” or less.	✓								
	Health literacy, performance-based: a brief comprehension test based on instructions similar to those found on a packet of aspirin bought over the counter [24]. Participants are asked to read a fictitious medicine label and respond to four questions, such as “What is the maximum number of days you may take this medicine?” and “List one condition for which you might take the tablet.” The task was developed according to a conceptual framework that defines literacy as an ability to fulfill goal-directed tasks, in this case, in a health context. Health literacy is categorized as high (maximum score), medium (one error), or low (more than one error).	✓								
	Digital literacy: the assessment of digital literacy was adapted from a validated instrument, the MACL^d^, a 7-point Likert scale consisting of statements about attitudes toward computers [25]. For the purpose of this study, the questions were modified to reflect the context of smartphone usage, such as “I can switch on and off a mobile phone.” Participants’ mobile phone locus of control was assessed to understand how well they believe that they have control over the outcomes of events of their smartphone usage [25].	✓								
**Primary outcomes: patient-reported outcomes**										
	Health literacy skills: the HLQ^e^, a multidimensional tool that measures health literacy across nine distinct conceptual domains, will be used. The median reliability of the HLQ domains is 0.88, with a range from 0.77 to 0.90 [26]. For the purpose of this study, we have selected three to domains to explore: Domain 2: have sufficient information to manage my health Domain 6: ability to actively engage with health care providers Domain 9: understand health information well enough to know what to do. Domain 2 assesses the strength of a participant’s agreement with the statement on a 4-point ordinal scale, ranging from 1 (“strongly disagree”) to 4 (“strongly agree”); domains 6 and 9 assess a participant’s perceived ease in task completion on a 5-point ordinal scale, ranging from 1 (“always difficult”) to 5 (“very easy”).	✓		✓		✓				✓
	Decision self-efficacy: the Decision Self-Efficacy Scale [23] measures self-confidence or belief in one’s ability to make decisions, including participation in shared decision-making. This item consists of 11 questions where participants rate their confidence engaging in decision-making behaviors, such as “Getting the facts about the medication choices available.” Items are rated on a 5-point ordinal scale ranging from 1 (“not at all confident”) to 5 (“very confident”).	✓		✓		✓				✓
	Rates of unscheduled health encounters: this includes unexpected hospital and emergency visits and unscheduled dialysis events in the past 3 months as reported by participants; this is also verified by extracting this information from electronic medical records.			✓		✓				✓
**Secondary outcomes: patient-reported outcomes**										
	Quality of life: the EQ-5D-5L^f^ is a standardized instrument used to measure health-related quality of life in a wide range of health conditions and treatment settings [27].	✓		✓		✓				✓
	Knowledge: an 8-item curriculum-based measure was purpose designed to assess knowledge about four key topics of dialysis self-management covered in the SUCCESS^g^ app: (1) diet, (2) fluids, (3) medicines, and (4) physical activity. Questions (two per topic) were based on key learnings from the app, with points scored for correct answers (0 = incorrect, 1 = correct).	✓		✓		✓				✓
	Health behavior**:** a theory-informed 11-item behavior questionnaire was adapted from previous literature [28] and matched to the content of the SUCCESS app. Participants are asked to respond “yes” or “no” to engaging in behaviors over the past week, such as “Checking the nutrition label when eating packaged food” (0 = no, 1 = yes).	✓		✓		✓				✓
	Confidence: an 11-item confidence measure was purpose designed based on the health behavior questionnaire above, including questions such as “How confident do you feel reading and understanding food labels?” Items are rated on a 5-point ordinal scale ranging from 1 (“not at all confident”) to 5 (“extremely confident”).	✓		✓		✓				✓
	Self-management: two questions were designed to assess patient knowledge and adherence to phosphate binder medication prescription, as an indicator of self-management.	✓		✓		✓				✓
**Secondary outcomes: clinical outcomes**										
	Changes in symptom burden: the POS^h^ is a widely used and validated instrument used in clinical care and research. The POS-Renal is a short measure (11 questions) that collects information on the most common symptoms that renal patients experience [29].	✓		✓		✓				✓
	Nutritional status: the PG-SGA^i^ is a standard nutritional assessment tool measuring clinical indices (ie, weight, intake, symptoms, functional status, disease state, metabolic stress, and nutritional physical examination) in a range of chronic conditions [30]. The PG-SGA enables the patient and professional to quickly and easily assess and monitor the risk for malnutrition and to evaluate effects of interventions.	✓		✓		✓				✓
	Interdialytic weight gain: this is a measure of weight gained between two consecutive dialysis sessions. The measure gives an indication of whether patients are adhering to fluid restrictions and is recorded at each dialysis session; the monthly average is easily calculated for each patient.	✓		✓		✓				✓
	Key performance indicators: typical key performance indicators for dialysis will be linked and extracted from the Australian and New Zealand Dialysis and Transplant registry. This will include demographics, ethnicity, anthropometry, comorbidities, course of treatment, and survival rates.	✓		✓		✓				✓
	App analytics—the following information will be extracted using Google Analytics: How many people have registered or logged in? How often do people log in? At what times? How does this relate to their dialysis times? How long do people stay logged in? Time between visits Number of times people access app features (eg, quizzes and videos) Number of visits to each page and subpage.	✓		✓		✓				✓

^a^A checkmark indicates that the measure will be performed at the indicated timepoint.

^b^MoCA: Montreal Cognitive Assessment.

^c^MMSE: Mini–Mental State Examination.

^d^MACL: Multicomponent Assessment of Computer Literacy.

^e^HLQ: Health Literacy Questionnaire.

^f^EQ-5D-5L: 5-level EQ-5D.

^g^SUCCESS: Supporting Culturally and Linguistically Diverse CKD Patients to Engage in Shared Decision-Making Successfully.

^h^POS: Palliative care Outcome Scale.

^i^PG-SGA: Patient-Generated Subjective Global Assessment.

### Data Collection and Management

Quantitative data will be either collected using paper copies or captured electronically via REDCap, a web-based tool to capture data for clinical research and to create databases and projects. Baseline data will be collected in person, and follow-up data will be collected either in person or electronically based on patient preference and depending on COVID-19 restrictions at the time of data collection.

After enrollment, a unique identifier will be assigned to each study participant. Personal information about participants will be kept separate from the main data set and will not be shared. All data will be stored on password-protected REDCap databases. Spreadsheets and data analysis derived from this data will be stored in the University of Sydney Research Data Store only, and will not be downloaded to individually owned computers.

### Qualitative Substudy

Qualitative data will be collected from participants at 3, 6, and 12 months. Semistructured interviews will be conducted by research staff trained in qualitative methods, in order to explore the experiences and perspectives of individual participants regarding usage of the app. The perspectives of users on the applicability of content and their ability to build capacity, become motivated, and identify opportunities to improve the management of their CKD will also be sought. Additional feedback from research participants on the content and design of the app will be discussed.

Participants for qualitative interviews will be purposively selected from those already enrolled to capture a range of demographic characteristics, including those with lower health literacy and from culturally and linguistically diverse backgrounds. Interviews will be conducted face-to-face during participants’ scheduled dialysis sessions or virtually via approved videoconferencing software. Participants dialyzing at home may complete interviews over the phone or virtually or can arrange a time to meet the interviewer at the dialysis clinic, depending on the COVID-19 restrictions at the time. All participants will have provided consent to participate in the interviews at the beginning of the study. Verbal consent will be confirmed for the interview, and the audio recording will be obtained at the start of the interview. The semistructured interviews are expected to last between 20 and 60 minutes and will be audio recorded.

### Data Analysis

#### Overview

Analysis of data will occur using an intention-to-treat approach and will compare the intervention and usual care arms. For continuous and binary outcomes, linear and logistic regression models will be used, respectively, with study arm as a covariate. All models will also be adjusted for center using a random effect, and the type of patient (ie, receiving home-based or in-center care) will be adjusted for using a covariate.

Qualitative data will be analyzed using the Framework approach to thematic analysis [31]. Framework analysis is a matrix-based approach with columns depicting themes and rows listing the cases, enabling the relationship between themes and cases to be explored. The first step involves familiarization with the data, where one or two researchers will review the transcript and, using an inductive approach, they will then develop a list of emerging topics and salient themes. These initial impressions will form the basis of the coding framework. Then, additional researchers will independently read a subset of transcripts and review the framework, which will further be revised with continuous discussion if needed. One researcher will then code all the interviews into the final framework, of which a random subset (10%) will be double coded by an additional researcher to ensure rigor. Similarities or differences will be discussed and reassessed. Microsoft Excel will be used to summarize the themes and supporting quotes from each transcript in the framework. Prominent themes arising from the framework will be identified and discussed in-depth with the research team.

#### Missing Data

We will use best practice to decide when imputation is necessary for missing data, except for the outcome data, considering the percentage of missing data and plausibility of the “missing at random” assumption [32]. Where there is a large proportion of missing data, we will consider using a complete case or pairwise deletion approach. We will also perform sensitivity analyses to evaluate the robustness of our results. For outcome data, we will not use imputation methods and will only exclude participants who do not have baseline data and at least one follow-up outcome measure.

### Sample Size

Our sample size has been calculated based on the primary outcome of the three Health Literacy Questionnaire (HLQ) domains. We assumed a minimal clinically important difference of 0.25 units in any one of the three HLQ domains. A sample size of 384 participants would result in greater than 90% power to detect a 0.25-unit change in an HLQ domain between the two arms, assuming an SD of 0.60, and at least 80% power, assuming an SD of 0.75. These SD values are based on published literature and equate to a standardized difference of 0.42 and 0.33, respectively; a standardized difference of 0.5 is considered a medium effect [33,34]. Our power calculation is based on conducting our analyses using 2-sided tests, a 5% significance level, and assuming 20% loss to follow-up (eg, due to death or kidney transplant).

For qualitative interviews, a maximum total sample size of 40 participants is planned. Interviews will continue until data saturation or until the maximum total sample size is reached.

### Safety and Monitoring

The research staff team will meet fortnightly with the trial coordinator, project lead, or both to discuss recruitment and ensure that the rights, safety, and well-being of the participants enrolled in the trial are protected. At these meetings, the trial coordinator will verify that study activities and documentation are compliant with the study protocol. The trial coordinator and the research assistants will report any issues to the site-specific principal investigator as they arise. Furthermore, the trial coordinator and research staff team will meet regularly with the site-specific principal investigators and the University of Sydney investigators to report on the trial progress. If a participant is found to experience distress during data collection, the research assistants will have resources available for participants to be referred to the Beyond Blue Support Line, Lifeline, or back to their treating clinicians should they require it.

### Accounting for Extenuating Circumstances

As per the CONSERVE (CONSORT and SPIRIT Extension for RCTs Revised in Extenuating Circumstances) 2021 statement [35], we have outlined our plan if the SUCCESS trial is interrupted due to COVID-19 restrictions (Textbox 2).

**Approach to deal with interruptions due to COVID-19 restrictions.**

**Extenuating circumstances**

During the COVID-19 pandemic, there have been several occasions when the Australian government has announced lockdowns to reduce the risk of COVID-19 transmission within the community. During these lockdowns, research activities have been put on hold to reduce the risk of COVID-19 exposure to both participants and health professionals. Unfortunately, it is difficult to predict when these lockdowns may occur.

**Impact**

COVID-19 lockdowns may result in a slower recruitment rate and higher rate of participants lost to follow-up due to the inability of research staff to collect data at dialysis centers. Missing data may also arise directly or indirectly due to COVID-19. Procedures for missing data have already been specified in the protocol.

**Mitigating strategies**

Although face-to-face is the preferred approach for data collection for this trial, ethical approval has also been obtained to conduct the trial electronically or virtually. In this case scenario, the blind version of the Montreal Cognitive Assessment will be used, and the Patient-Generated Subjective Global Assessment will be collected at a later timepoint. Therefore, to reduce the interruptions due to COVID-19 lockdowns, the trial will be conducted electronically or virtually.

### Ethics Approval

Ethical approval for this study was granted by the Nepean Blue Mountain Local Health District Ethical Committee as well as the local research governance offices at each of the participating sites (2020/ETH00910).

## Results

Recruitment has begun at nine sites. We expect to finalize data collection by 2023 and publish the manuscript by 2024.The results from the RCT will be submitted for publication to an international peer-reviewed journal, regardless of the findings. In addition, the findings will be disseminated at conferences, and updates will be shared with partners, including participating local health districts, NSW Health, Kidney Health Australia, and consumers. The SUCCESS app will continue to be available to all participants undertaking the trial, after the trial has ended. In addition, the SUCCESS app will also be publicly available on the Apple App Store and Google Play for anyone to download.

## Discussion

This pragmatic RCT, which will be implemented within a real-world setting, aims to determine the effectiveness of a complex health intervention—the SUCCESS app—to improve patient-reported psychosocial and clinical outcomes. Results from previous research by our group suggest that our recruitment strategy will lead to a diverse population, including people from culturally and linguistically diverse backgrounds [16,36] and those with varying levels of cognition and health literacy [37]. In this way, the trial seeks to address limitations of health literacy research conducted to date.

A key strength of this study is that the SUCCESS app is designed to develop transferable skills in health literacy and shared decision-making in a group of patients who have complex needs. That the app has not yet been translated into other languages limits the selection of participants into the trial and the generalizability of findings, and COVID-19 lockdowns across Sydney continue to impact recruitment, data collection, and study timelines.

The management of CKD requires constant patient involvement based on very complex and often difficult-to-understand health advice. If the SUCCESS app is effective, these skills could be applied across multiple decision contexts, such as CKD stages III and IV and those under renal supportive care, and could extend to the management of comorbidities associated with kidney failure. This is a particular strength, given that the majority of patients with CKD have comorbidities, such as cardiac disease, cerebrovascular disease, diabetes, and lipid disorders.

## Appendix

Multimedia Appendix 1 External peer review report from the New South Wales (NSW) Ministry of Health - Translational Research Grants Scheme (TRGS) - TRGS EOI Review Panel (Australia).

## Abbreviations

CKD: chronic kidney disease
CONSERVE: CONSORT and SPIRIT Extension for RCTs Revised in Extenuating Circumstances
CONSORT: Consolidated Standards of Reporting Trials
HLQ: Health Literacy Questionnaire
PG-SGA: Patient-Generated Subjective Global Assessment
RCT: randomized controlled trial
REDCap: Research Electronic Data Capture
SPIRIT: Standard Protocol Items: Recommendations for Interventional Trials
SUCCESS: Supporting Culturally and Linguistically Diverse CKD Patients to Engage in Shared Decision-Making Successfully

## Data Availability

The data sets generated and analyzed during this study will be available from the corresponding author on reasonable request.
