# Supplementary material for: Evaluation of the SUCCESS Health Literacy App for Australian Adults With Chronic Kidney Disease: Protocol for a Pragmatic Randomized Controlled Trial
Source: JMIR Res Protoc. 2022 Aug 31;11(8):e39909. doi: 10.2196/39909 (PMC9475407; doi:10.2196/39909)
Supplement: Multimedia Appendix 1 [file resprot_v11i8e39909_app1.pdf]

NSW GOVERNMENT | Health

Associate Professor Kamal Sud
Chief Investigator
Nepean Blue Mountains Local Health District

TRIM: H18/111072

Dear Associate Professor Sud,

Re: Translational Research Grants Scheme Round 4

I am delighted to inform you that your Expression of Interest for funding under the Translational Research Grants Scheme (TRGS) Round 4 for the project: 'Supporting adults with CKD to engage in shared decision making successfully (SUCCESS): Phase III Pilot Study' (TRGS Application Number: 31) has been selected to progress to the Full Application stage.

A total of 74 Expressions of Interest were received this round. All Expressions of Interest were assessed through a competitive process by the TRGS EOI Review Panel with advice from an internal Sub-Committee and other relevant experts where required. The Review Panel selected approximately 25% of Expressions of Interest to progress to Full Application. Your proposal now stands a good chance of being granted funding.

I am pleased to invite you to develop and submit a Full Application for consideration by the TRGS Selection Panel. Your Full Application should be prepared on the form provided. The focus of the Full Application is a detailed research plan describing how the proposed project will be implemented.

To assist in the development of the Full Application, feedback from the TRGS EOI Review Panel is provided as an attachment to this letter. Please ensure that this feedback is addressed in your Full Application, and your budget request is adjusted as required to satisfactorily address the Review Panel's comments. If you would like to discuss the feedback in more detail, please contact MOH-TRGS@health.nsw.gov.au, quoting the TRGS Application Number above, to arrange a suitable time to speak.

The Full Application must be approved by the Chief Executives of all Local Health Districts and Specialty Health Networks involved as partners in the project. The Full Application must be submitted by the TRGS Coordinator of your Host Organisation by email by **5pm Wednesday 13 March, 2019**. Other instructions for applicants are included in the Full Application form. Full Applications will be assessed by the TRGS Selection Panel, according to the selection criteria as set out in the TRGS Guidelines for Applicants (available at: https://www.medicalresearch.nsw.gov.au/translational-research-grants-scheme/).

I would like to take this opportunity to congratulate you on the success of your Expression of Interest for TRGS funding and commend you on your commitment to research that will contribute to improving the health of the people of NSW.

Yours sincerely

K Chant

Dr Kerry Chant PSM
Chief Health Officer and Deputy Secretary
Population and Public Health

TRGS Round 4 Expression of Interest: Feedback from the TRGS EOI Review Panel

TRGS Application Number:	31
Chief Investigator:	Associate Professor Kamal Sud
Host Organisation:	Nepean Blue Mountains Local Health District
Project Title:	Supporting adults with CKD to engage in shared decision making successfully (SUCCESS): Phase III Pilot Study

The extent to which the project will generate new and relevant evidence for policy and/or practice; and likely impact of the results of the project on disease prevention, patient care or health service delivery:

This is an important area for further research, and a feasibility study has already been conducted.

Data from the feasibility study should be provided to strengthen the research proposal.

The title and wording should be changed to remove the references to 'Phase III' as it may be confused with a Phase III clinical trial.

The reviewers noted that Aboriginal people are not included in the research proposal. Aboriginal people experience a disproportionate burden of CKD:

- They are more than twice as likely as non-Aboriginal people to have indicators of CKD, and
- They account for approximately 9 per cent of people commencing kidney replacement therapy each year

It is recommended that the research proposal highlight the burden of CKD faced by Aboriginal people and how the study will be culturally appropriate and culturally safe for Aboriginal people.

In addition, the study should include Aboriginal people as one of the target population groups.

Strength, rigour and appropriateness of research design:

The study should aim to measure the impact of health literacy on appropriate care and patient outcomes. These should be included in the study design and outcome measures.

Consideration should be given to including a comparison or control group, or whether a stepped wedge design would be appropriate.

Further detail is needed for data sources, outcome measures, statistical analysis, and sample size, effect size and power calculations.

Further detail is required about measurement of costs and cost-effectiveness analysis.

Use of Respecting the Difference training should be considered for supporting the cultural competency of any health professionals delivering health services or undertaking research with Aboriginal people.

Ability of research team to carry out proposed project within timeframe:

It is recommended that an advisory group be established for this research, and that there be broader engagement eg with St George Hospital and Hunter New England LHD.

The research team does not include partnership with Aboriginal health organisations. Given the burden of disease, it is recommended that an ACCHS (e.g. Greater Western Aboriginal Health Service) should be included as a partner and/or on the advisory group for this project. The local Aboriginal Health Unit should also be involved and provided with the opportunity to participate.

The Centre for Aboriginal Health, NSW Ministry of Health, should also be included as a partner.

Extent to which project supports research translation:

The proposal states that the research is at the effectiveness and scalability stage, however, it is more likely at the efficacy stage.

Further detail is needed of how research findings might be implemented, including engagement with key stakeholders, dissemination activities and publication of findings.

Budget:

TRGS funding requests for service delivery components of a project need to be carefully considered. The TRGS has agreed to fund service delivery in many instances if it is necessary for the research to be performed, as long as the relevant Chief Executive agrees to continue funding on an ongoing basis if positive findings are demonstrated. Where the intervention is potentially resource intensive the project should plan for a full cost-effectiveness analysis pending positive findings.

Other comments:
It is strongly recommended that the research team schedule a follow up call with a review panel member to discuss feedback prior to the development of the Full Application. To schedule this call, email the TRGS team at MOH-TRGS@health.nsw.gov.au.
